# Usefulness of the BACES score in nontuberculous mycobacterial pulmonary disease for various clinical outcomes

**DOI:** 10.1038/s41598-023-33782-z

**Published:** 2023-05-09

**Authors:** Hyung-Jun Kim, Myung Jin Song, Byoung Soo Kwon, Yeon Wook Kim, Sung Yoon Lim, Yeon-Joo Lee, Jong Sun Park, Young-Jae Cho, Choon-Taek Lee, Jae Ho Lee

**Affiliations:** 1 Division of Pulmonary and Critical Care Medicine, Department of Internal Medicine, Seoul National University Bundang Hospital, Seongnam, Republic of Korea; 2 Department of Internal Medicine, Seoul National University College of Medicine, Seoul, Korea

**Keywords:** Risk factors, Infectious diseases, Respiratory tract diseases

## Abstract

A uniform prognostic marker is needed for nontuberculous mycobacterial pulmonary disease (NTM-PD) due to the diverse clinical course. We aimed to seek the utility of the BACES score, originally derived to predict all-cause mortality, for various outcomes. To calculate the BACES score, one point was given for each of the following factors: body mass index < 18.5 kg/m^2^, age ≥ 65 years, presence of cavities, elevated erythrocyte sedimentation rate, or male sex. The study included 681 patients, of whom 97 (14.2%), 189 (27.7%), 192 (28.2%), 143 (21.0%), 47 (6.9%), and 13 (1.9%) had BACES scores of 0 to 5. Those with greater BACES scores were more likely to experience dyspnea, body weight loss, and anorexia. With severe disease, the risk of disease progression increased while the rate of treatment culture conversion decreased. After adjustment of comorbidities, higher BACES score was independently associated with the risk of mortality from respiratory causes or infection. As a simple and efficient bedside tool for assessing the severity of NTM-PD, the BACES score has the potential to be utilized as a surrogate marker for uniform severity assessment.

## Introduction

Nontuberculous mycobacteria (NTM) are ubiquitous, opportunistic pathogenic bacteria in the environment, such as soil and water^1^. NTM can cause chronic progressive lung disease known as a non-tuberculous mycobacterial pulmonary disease (NTM-PD)^2,3^. The prevalence of NTM-PD is increasing worldwide^4–6^, and its overall mortality has been emphasized in several recent studies^7–9^. A previous study conducted in the United States reported that the 5-year mortality rate of NTM-PD was 25.0%, even without any comorbidities, which was higher than a rate of 5.7% in the general population^9^.

NTM-PD has a broad range of clinical courses. Spontaneous negative culture conversion can occur in approximately 40% of patients with non-cavitary bronchiectasis^10^; the remaining patients may experience an aggravation of the disease, leading to respiratory failure and death^11^. It is difficult to predict the clinical course upon diagnosis; earlier treatment is considered in those with positive smear results or cavitary lesions, whereas “watchful waiting” recommended as an alternative to immediate treatment in other patients^2,3,12^.

Recently, a simple numerical model was developed comprising five clinical variables: body mass index (BMI), age, presence of cavity, elevated erythrocyte sedimentation rate (ESR), and sex^13^. The scoring system was named the BACES score; it was developed and validated in two different centers in South Korea and shown to predict all-cause mortality with excellent performance^13^. The BACES score was also associated with the culture conversion rate after treatment in the same population^14^. The BACES score needs to be validated in a different patient population to widen its use^15,16^. In this study, we aimed to discuss various utilities of the BACES score in other groups of patients with NTM-PD considering multiple clinical aspects.

## Methods

### Patient selection and data collection

Patients diagnosed with NTM-PD at Seoul National University Bundang Hospital between May 1, 2003, and April 30, 2018, were included in this cohort study. Patients’ data were reviewed to determine whether the diagnostic criteria for NTM-PD were met^2,3,17^. We excluded patients who had previously received antibiotic therapy for NTM-PD, patients with insufficient data regarding the BACES score, or who were not followed up after diagnosis.

Patient demographics, smoking history, symptoms, comorbidities, and chest computed tomography (CT) findings were retrieved from the electronic medical records. The date of NTM-PD diagnosis was defined as the date of the second culture-positive sputum collection or the date of culture-positive bronchoscopy specimen collection^18^. In contrast to the previous studies^13,19^, all NTM species, including *Mycobacterium avium* complex (MAC), *Mycobacterium abscessus* complex (MABC), and *Mycobacterium kansasii,* were included. When a different NTM species, not the one detected initially, was seen at least twice during follow-up, the infection was considered a mixed infection^18,20^.

The Institutional Review Board of Seoul National University Bundang Hospital (B-2203–743-102) approved this study and waived the requirement for informed consent due to the use of anonymized data and the study's retrospective nature. This study was conducted according to the principles of the Declaration of Helsinki.

### Calculation of the BACES score

The BACES score is composed of five variables: BMI < 18.5 kg/m^2^, age ≥ 65 years, presence of a cavity on chest CT, elevated ESR (men > 15 mm/h, women > 20 mm/h), and male sex^13^. Chest CT findings and ESR within a year of diagnosis of NTM-PD but before treatment of the disease were used. Each variable was assigned one score, with a minimum score of 0 and a maximum score of 5. The estimated probabilities of 5-year mortality according to the BACES scores are reported as 1.2% for a score of 0, 3.2% for a score of 1, 8.4% for a score of 2, 21.3% for a score of 3, 47.8% for a score of 4, and 82.9% for a score of 5^13^. The patients were divided into three groups as follows: the mild (BACES score 0–1), moderate (BACES score 2–3), and severe (BACES score 4–5) groups^14^.

### Outcomes

Progression of NTM-PD was defined as the initiation of treatment for the disease^21–23^. Owing to the retrospective nature of the study, there was no uniform protocol for treating NTM-PDs, and attending physicians referred to the most recent American Thoracic Society/The Infectious Diseases Society of America/British Thoracic Society guidelines for appropriate treatment of the identified NTM species^2,3,17^. Culture conversion was defined as at least three consecutive negative sputum cultures after treatment. The day of collection of the first culture-negative sputum was considered the day of negative culture conversion^24^. The date and leading cause of death were collected from death certificates described by the attending physicians and confirmed using the Statistics Korea database using the Korean Standard Classification of Diseases, 7th edition, until December 31, 2020. Because there is no solid definition of NTM-PD-associated mortality^8^, two investigators (HJK and JHL) examined the causes of death associated with the respiratory system or infection were selected. Any discrepancies were resolved through group discussions. Details of the causes of death are listed in Supplementary Table S1.

### Other statistical considerations

Categorical variables are expressed as numbers (percentiles) and *p*-values were calculated using the chi-square or Fisher’s exact test. In contrast, continuous variables are expressed as median (interquartile range [IQR]), and *p* values were calculated using the Kruskal–Wallis test. For survival analyses, Kaplan–Meier curves were drawn, and *p* values were calculated using the log-rank test with Bonferroni adjustment for multiple comparisons. A Cox regression analysis was performed to calculate the hazard ratio (HR) with 95% confidence intervals (CIs). The *p* values of < 0.05 were considered statistically significant. Our observed data were utilized to calculate the 5-year probability of all-cause mortality using the Kaplan–Meier estimator.

## Results

### Baseline characteristics

Six hundred eighty-one patients were included in this study (Supplementary Figure S1). Most of the patients were female (56.2%), with a median age of 66.0 (interquartile range [IQR], 56.7–73.2) years and a BMI of 21.1 (IQR, 19.2–23.1) kg/m^2^. The most prevalent comorbidity was a history of tuberculosis (31.1%), followed by hypertension (24.4%), malignancy (17.3%), and diabetes mellitus (13.2%). Most of the patients had a mild-to-moderate severity of NTM-PD; 97 (14.2%), 189 (27.7%), 192 (28.2%), 143 (21.0%), 47 (6.9%), and 13 (1.9%) patients had BACES scores of 0, 1, 2, 3, 4, and 5, respectively (Supplementary Figure S2).

Patients with severe NTM-PD were more likely to have a smoking history (51.6%) than those with mild (11.2%) and moderate NTM-PD (34.1%) (*p* < 0.001 by chi-square test). A history of tuberculosis and chronic pulmonary comorbidities, such as chronic obstructive pulmonary disease, asthma, and idiopathic pulmonary fibrosis, were more common in patients with severe NTM-PD than in others. Compared to patients with moderate (71.6%) and mild (61.9%) NTM-PD, patients with severe NTM-PD were more likely to be diagnosed using two repetitive sputum cultures (83.3%) rather than invasive methods (*p* = 0.001 by chi-square test). Patients with severe NTM-PD also had a higher initial sputum smear positivity rate (36.7% vs. 10.5% vs. 6.6%, *p* < 0.001 by chi-square test) (Table 1).

**Table 1:** Baseline characteristics of NTM-PD patients.

Variables	Overall	BACES 0–1	BACES 2–3	BACES 4–5	*p*
	N = 681	n = 286	n = 335	n = 60	
Sex, female	383 (56.2)	237 (82.9)	143 (42.7)	3 (5.0)	< 0.001
Age, years	66.0 [56.7–73.2]	58.6 [52.4–64.0]	70.1 [64.2–75.9]	73.0 [68.0–77.3]	< 0.001
BMI, kg/m^2^	21.1 [19.2–23.1]	21.6 [20.0–23.6]	20.9 [19.0–22.9]	18.1 [15.9–20.2]	< 0.001
Smoking history					
Never smoker	504 (74.0)	254 (88.8)	221 (66.0)	29 (48.3)	< 0.001
Former smoker	141 (20.7)	22 (7.7)	96 (28.7)	23 (38.3)	
Current smoker	36 (5.3)	10 (3.5)	18 (5.4)	8 (13.3)	
Comorbidities					
History of tuberculosis	212 (31.1)	69 (24.1)	110 (32.8)	33 (55.0)	< 0.001
Hypertension	166 (24.4)	48 (16.8)	103 (30.7)	15 (25.0)	< 0.001
Malignancy	118 (17.3)	29 (10.1)	76 (22.7)	13 (21.7)	< 0.001
Diabetes mellitus	90 (13.2)	18 (6.3)	59 (17.6)	13 (21.7)	< 0.001
Asthma	64 (9.4)	27 (9.4)	34 (10.1)	3 (5.0)	0.453
COPD	59 (8.7)	3 (1.0)	41 (12.2)	15 (25.0)	< 0.001
Cardiovascular disease	49 (7.2)	15 (5.2)	31 (9.3)	3 (5.0)	0.123
Neuromuscular disease	45 (6.6)	13 (4.5)	23 (6.9)	9 (15.0)	0.012
IPF	11 (1.6)	0 (0.0)	10 (3.0)	1 (1.7)	0.013
Chronic liver disease	10 (1.5)	0 (0.0)	8 (2.4)	2 (3.3)	0.022
Diagnostic method					
Sputum	467 (68.6)	177 (61.9)	240 (71.6)	50 (83.3)	0.001
Bronchoscopy	183 (26.9)	88 (30.8)	85 (25.4)	10 (16.7)	
Tissue biopsy	31 (4.6)	21 (7.3)	10 (3.0)	0 (0.0)	
Positive sputum smear	76 (11.2)	19 (6.6)	35 (10.5)	22 (36.7)	< 0.001
Presence of cavity	135 (19.8)	14 (4.9)	77 (23.0)	44 (73.3)	< 0.001
Erythrocyte sedimentation rate, mm/h	19.0 [9.0–36.0]	11.0 [6.0–19.0]	27.0 [13.0–45.0]	37.0 [26.0–53.0]	< 0.001
Duration of follow-up, years	4.9 [1.8–8.5]	6.2 [2.4–9.8]	4.8 [1.7–8.0]	1.8 [0.8–3.3]	< 0.001

*NTM-PD* nontuberculous mycobacterial pulmonary disease; *BMI* body mass index; *IPF* idiopathic pulmonary fibrosis; *COPD* chronic obstructive pulmonary disease.

Numbers are presented as count (percentage) or median [interquartile range]. The *p* values were calculated using the chi-square or Fisher’s exact test for categorical variables, or the Kruskal–Wallis test for continuous variables.

### Patient symptoms and NTM species

Common symptoms were cough (36.0%), sputum (29.1%), hemoptysis (18.2%), and dyspnea (13.8%). Patients with severe NTM-PD were more likely to have dyspnea (26.7%) than those with mild (8.4%) and moderate (16.1%) NTM-PD (*p* < 0.001 by chi-square test). Symptoms of body weight loss (6.7% vs. 3.0% vs. 1.0%, *p* = 0.029 by chi-square test) and anorexia (3.3% vs. 0.3% vs. 0.0%, *p* = 0.002 by chi-square test) were also more common in patients with higher BACES scores (Supplementary Table S2).

MAC was the prevalent causative species (68.8%), followed by MABC (14.5%) and *Mycobacterium kansasii* (2.5%). Fifty-five (8.1%) patients were diagnosed with mixed infections. Patients with higher BACES scores had were more likely to have *M. intracellulare* and *M. abscessus* infections. In contrast, those with lower BACES scores were more likely to have *M. avium* and *M. massiliense* infections (*p* = 0.008 by chi-square test) (Supplementary Table S3).

### Disease progression and treatment response

**Figure 1:**
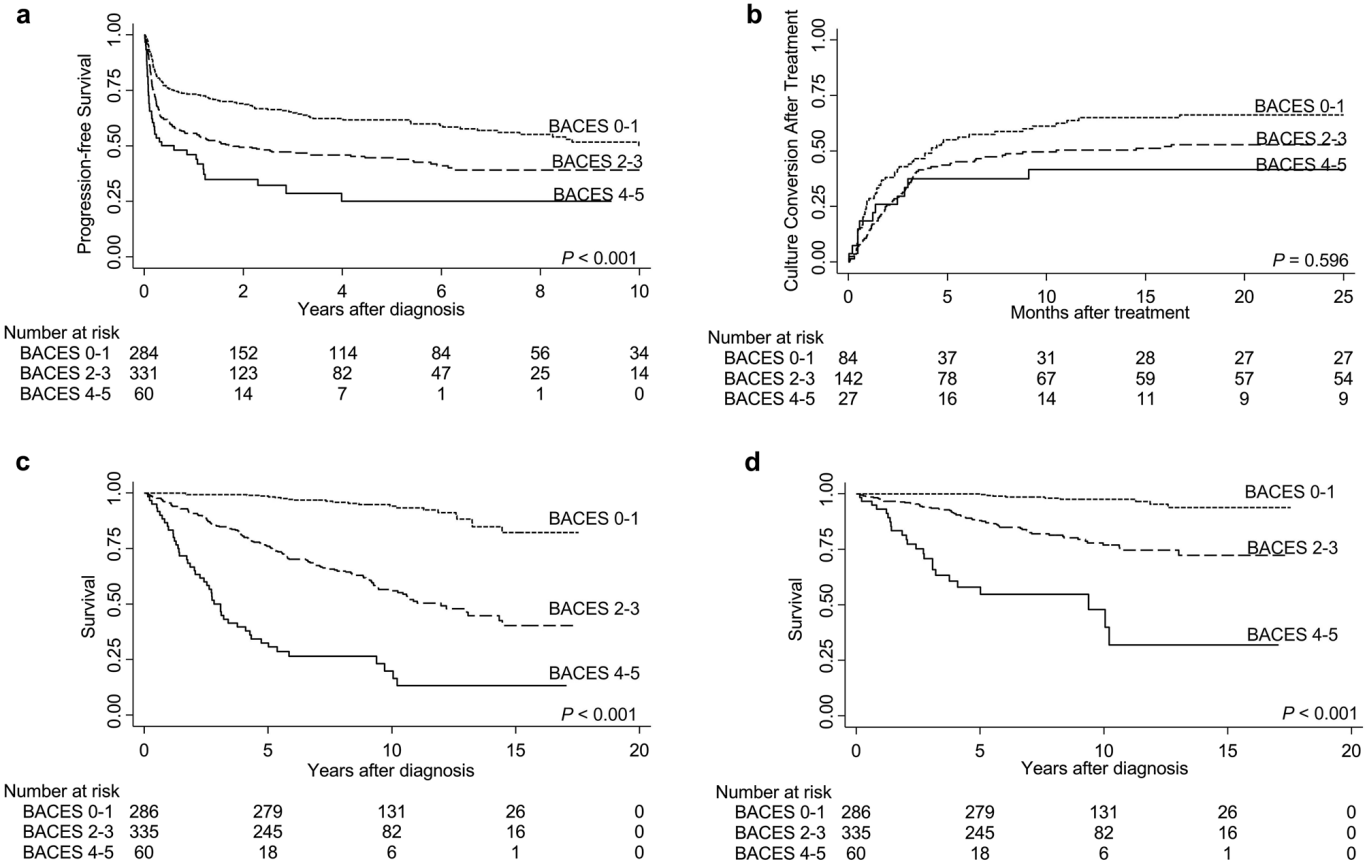
Patient outcomes after diagnosis of NTM-PD. Patients with higher BACES scores have a lower probability of progression-free survival (**a**) and negative culture conversion after treatment (**b**). The probabilities of all-cause mortality (**c**) and mortality from respiratory cause or infection (**d**) were higher with a higher BACES score. *P* values were calculated using the log-rank test with Bonferroni adjustment for multiple comparisons. Abbreviation: NTM-PD, nontuberculous mycobacterial pulmonary disease.

Of the 681 patients, 333 (48.9%) had progressive disease. The median time to progression was 2.6 (IQR, 1.2–10.5) months, which was shorter for patients with severe NTM-PD (median, 1.3 months) than for those with moderate (2.5 months) or mild (3.8 months) NTM-PD (*p* < 0.001). Compared to patients with mild NTM-PD, patients with moderate (HR, 1.66; 95% confidence interval [CI], 1.31–2.11) or severe (HR, 2.76; 95% CI, 1.91–3.99) NTM-PD had a higher risk of disease progression (log-rank test with Bonferroni correction *p* < 0.001) (Table 2 and Fig. 1a). Of the five components of the BACES score, the presence of any cavity (HR, 2.25; 95% CI, 1.77–2.88), low BMI (HR, 1.76; 95% CI, 1.35–2.30), elevated ESR (HR, 1.59; 95% CI, 1.27–1.98), and male sex (HR, 1.27; 95% CI, 1.02–1.58) were associated with the risk of disease progression. Elderly age (≥ 65 years) was not significantly associated with disease progression (HR, 1.05; 95% CI, 0.85–1.31).

**Table 2:** Clinical outcomes after diagnosis of nontuberculous mycobacterial pulmonary disease.

Variables	Overall	BACES 0–1	BACES 2–3	BACES 4–5	*p*
	N = 681	n = 286	n = 335	n = 60	
Disease progression					
Yes	333 (48.9)	115 (40.2)	179 (53.4)	39 (65.0)	< 0.001
Months to progression after diagnosis	2.6 [1.2–10.5]	3.83 [1.8–25.7]	2.53 [1.2–6.9]	1.3 [0.7–7.3]	< 0.001
Culture conversion after treatment*	142/256 (55.5)	55/84 (65.5)	76/145 (52.4)	11/27 (40.7)	0.042
Mortality					
Follow-up duration, years	8.1 [5.4–10.9]	9.5 [7.3–12.1]	7.1 [4.7–10.0]	2.8 [1.4–5.4]	0.001
All-cause	209 (30.7)	22 (7.7)	140 (41.8)	47 (78.3)	< 0.001
Due to respiratory causes or infection	94 (13.8)	9 (3.1)	61 (18.2)	24 (40.0)	< 0.001

*Calculated among 256 patients with ≥ 3 sputum cultures after treatment. Numbers are presented as count (percentage) or median (interquartile range) unless specified otherwise.

Of the 333 patients with progressive disease, 256 had at least three sputum cultures after treatment. Of the 256 patients, 142 (55.5%) had culture conversion. The culture conversion rate was 65.5% in mild NTM-PD, 52.4% in moderate NTM-PD, and 40.7% in severe NTM-PD (*p* = 0.042 by chi-square test). However, the time to culture conversion did not reveal a significant difference between the groups (log-rank test with Bonferroni correction *p* = 0.596) (Table 2 and Fig. 1b).

### Mortality

As of December 31, 2020, 209/681 (30.7%) patients had died after a median of 8.6 (IQR, 5.4–10.9) years following NTM-PD diagnosis. Compared to the mild group, the moderate (HR, 7.44; 95% CI, 4.74–11.67) and severe (HR 26.05, 95% CI 15.60–43.50) groups had higher risks of all-cause mortality in this separate cohort (log-rank test with Bonferroni correction *p* < 0.001) (Table 2 and Fig. 1c). The estimated survival rates and actual survival rates showed a good correlation (Supplementary Figure S3). Elderly age (HR, 6.51; 95% CI, 4.48–9.45), elevated ESR (HR, 3.54; 95% CI, 2.60–4.84), male sex (HR, 3.36; 95% CI, 2.52–4.48), lower BMI (HR, 2.33; 95% CI, 1.71–3.18), and presence of cavity (HR, 1.78; 95% CI, 1.31–2.42) were all associated with the time to all-cause mortality. Using the Kaplan–Meier estimator, the estimated probability of 5-year mortality from our observed data was as follows: BACES 0 (2.8%), BACES 1 (6.5%), BACES 2 (22.7%), BACES 3 (47.4%), BACES 4 (73.7%), and BACES 5 (91.0%).

**Figure 2:**
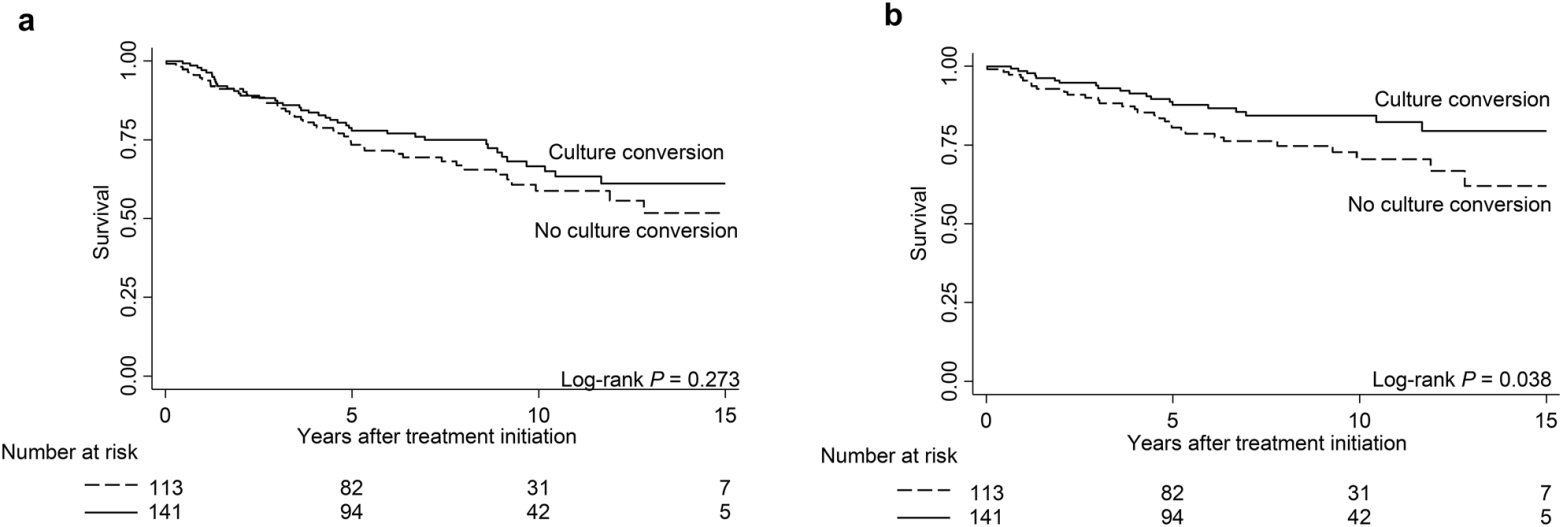
Patient survival according to the achievement of culture conversion. The graphs show Kaplan–Meier curves according to the achievement of ≥ 3 consecutive negative culture conversions after treatment. Solid lines indicate achievement of culture conversion, while dashed lines indicate failure to achieve culture conversion. Achievement of negative culture conversion was not associated with all-cause mortality (**a**) but with mortality from respiratory cause or infection (**b**).

Of 209 patients, 94 died of respiratory causes or infections. Details of the causes of death are presented in Supplementary Table S4. Patients with negative culture conversion had a lower mortality risk due to respiratory causes or infection (log-rank *p* = 0.038) (Fig. 2). Compared to the mild group, the moderate (HR, 7.80; 95% CI, 3.87–15.73) and severe (HR, 31.97; 95% CI, 14.73–69.42) groups had significantly higher risks of mortality from respiratory causes or infection (log-rank *p* < 0.001) (Table 2 and Fig. 1d). Elderly age (HR, 5.86; 95% CI, 3.42–10.05), elevated ESR (HR, 4.59; 95% CI 2.80–7.54), lower BMI (HR, 3.65; 95% CI, 2.39–5.58), male sex (HR, 2.36; 95% CI, 1.56–3.56), and the presence of a cavity (HR, 2.20; 95% CI, 1.42–3.41) were all associated with mortality from respiratory causes or infection.

Because the BACES score does not include underlying comorbidities, we performed a multivariate analysis including the underlying comorbidities. Compared to mild NTM-PD, moderate (adjusted HR, 5.13; 95% CI, 2.48–10.59) and severe (adjusted HR, 18.29; 95% CI, 7.92–42.20) NTM-PD were independently associated with the risk of mortality from respiratory causes or infection. Among the comorbidities, chronic liver disease (adjusted HR, 3.82; 95% CI, 1.23–11.90), tuberculosis (adjusted HR, 1.61; 95% CI, 1.05–2.47), chronic obstructive pulmonary disease (adjusted HR, 1.61; 95% CI, 1.75–4.64), and idiopathic pulmonary fibrosis (adjusted HR, 13.77; 95% CI, 6.26–30.31) were also independently associated with a higher risk (Table 3).

**Table 3:** Factors associated with mortality due to respiratory cause or infection in NTM-PD patients.

Variable	Unadjusted HR	Adjusted HR
BACES*		
0–1	Reference	Reference
2–3	7.80 (3.87 − 15.73)	5.13 (2.48 − 10.59)
4–5	31.97 (14.73 − 69.42)	18.29 (7.92 − 42.2)
Diabetes mellitus	2.09 (1.27 − 3.42)	1.42 (0.81 − 2.47)
Chronic kidney disease	3.16 (1.16 − 8.61)	0.95 (0.27 − 3.31)
Neuromuscular disease	1.93 (1.00 − 3.73)	1.11 (0.53 − 2.31)
Hypertension	1.92 (1.26 − 2.92)	1.45 (0.91 − 2.31)
Chronic liver disease*	8.42 (3.04 − 23.28)	3.82 (1.23 − 11.90)
Bronchiectasis	0.45 (0.30 − 0.68)	0.90 (0.57 − 1.41)
History of tuberculosis*	2.08 (1.39 − 3.13)	1.61 (1.05 − 2.47)
COPD*	5.94 (3.77 − 9.38)	2.85 (1.75 − 4.64)
IPF*	22.27 (11.26 − 44.04)	13.77 (6.26 − 30.31)

*NTM-PD* nontuberculous mycobacterial pulmonary disease; *HR* hazard ratio; *COPD* chronic obstructive pulmonary disease; *IPF* idiopathic pulmonary fibrosis.

Numbers are presented as hazard ratios with 95% confidence intervals.

*Statistically significant after multivariate adjustment (*p* < 0.05).

## Discussion

This is the first study to validate the BACES score for various clinical aspects of NTM-PD in a separate cohort. With a higher BACES score, the prevalence of symptoms such as dyspnea, anorexia, and body weight loss was higher. The risk of disease progression increased with a higher BACES score, but the probability of negative culture conversion was lower after treatment. In addition to all-cause mortality, the BACES score was independently associated with mortality from respiratory causes or infections, even after adjusting for underlying comorbidities. The strengths of this study include the different patient groups, broader species of NTM, and a longer follow-up duration until death (median, 8.1 years) compared to those in a similar previous study (median, 6.8 years and 4.9 years for the derivation and validation groups, respectively)^13^.

Our results demonstrate that the BACES score is an integrated score comprising core prognostic components, which have been identified repeatedly in earlier studies. A lower BMI is associated with a higher prevalence of NTM-PD and a higher risk of all-cause and MAC-specific mortality^25,26^. Older age is a risk factor for all-cause mortality^19,26–30^. The presence of cavities and elevated inflammatory markers are associated with a higher risk of radiographic deterioration, all-cause mortality, and MAC-specific mortality^26,28,29^. Men are more likely to die with NTM-PD than women^19,26,27^.

Initially, the BACES score was used to predict all-cause mortality in patients with NTM-PD^13^. In our study, the BACES score was also associated with a higher probability of disease progression, defined as the initiation of treatment. Although recent guidelines recommend initiating therapy for cavitary disease and positive smear results^3,12^, there is no definite standard to begin treatment, and shared decision-making is critical^15^. An objective measure of disease severity can be helpful in such circumstances, and the BACES score can be a valid parameter. In addition, the BACES score was associated with the probability of culture conversion after treatment, which was also observed in the original derivation cohort^14^.

We confirmed the association between the BACES score and mortality from respiratory causes or infection, even after adjustment for comorbidities. Patients are more likely to die “of” NTM-PD rather than “with” NTM-PD as the prevalence of NTM-PD increases^9^. However, given the high prevalence of comorbidities, it was challenging to identify the NTM-PD-specific deaths. As indicated in our study, patients with severe NTM-PD have a higher prevalence of underlying chronic lung diseases such as asthma, interstitial pulmonary fibrosis, and chronic obstructive pulmonary disease, which are essential factors influencing NTM-PD prognosis^26,27,29,30^. Although such chronic diseases affect survival, the BACES score is an independent predictor of mortality from respiratory causes or infection. This increases the scientific power of the BACES score as a prognostic marker in NTM-PD, regardless of the underlying medical conditions.

Negative culture conversion was associated with fewer deaths from respiratory causes or infections but not all-cause mortality. This differs from a previous study in which negative culture conversion observed within 6 to 12 months was associated with lower all-cause mortality^14^. Our study may be underpowered, considering that only 256 patients were included in the survival analysis of culture conversion, compared to approximately 700 patients in the original cohort^14^. Nonetheless, our results indicate that mortality from respiratory causes or infection can be reduced while eradicating NTM. We suggest that achieving culture conversion is essential regardless of the time to culture conversion. Taken together, patients with a higher BACES score had a higher risk of disease progression following treatment initiation, while the culture conversion rate was lower. Given the association between the achievement of culture conversion and better survival, those with a higher BACES score are less likely to survive.

This study has a few limitations. First, this study was conducted in South Korea, where the BACES score was developed. Further validation studies in other geographic areas and ethnicities are required. Second, due to the study’s retrospective design, the treatment protocols could not be predefined. However, the overall rate of negative culture conversion was 54.4% in MAC-PD, 51.7% in MABC-PD, and 87.5% in *Mycobacterium kansasii*-PD, which is comparable with the findings from previous reports^31–33^. Thirdly, while the observed mortality rate was higher than that of the derivation cohort^13^, the proportion of patients with subjective symptoms was lower than that reported in a recent study^34^. This may be due to the older age of our patients compared to the original cohort (median 66 vs. 60 years) and because we did not use a detailed questionnaire-based symptom assessment, the attending physician may not have noticed mild or intermittent symptoms like cough, sputum, or dyspnea.

## Conclusions

The BACES score is a simple integrated system associated with various clinical aspects. The BACES score might be a potential marker for disease progression, treatment response, death associated with the respiratory system or infection, and all-cause mortality. In addition to the recent recommendation of initiating treatment in patients with positive acid-bacilli smears and/or cavitary diseases^12^, those with a higher BACES score can receive aggressive treatment including intravenous antibiotics, rather than “watchful waiting.” Further studies are needed to determine whether aggressive therapy for patients with a higher BACES score is associated with better outcomes.

## Supplementary Information

Supplementary Information.

## Data Availability

The data supporting this study’s findings are available from the corresponding author upon reasonable request. The data are not publicly available due to privacy or ethical restrictions.
