# Supplementary material for: Usefulness of the BACES score in nontuberculous mycobacterial pulmonary disease for various clinical outcomes
Source: Sci Rep. 2023 May 9;13:7495. doi: 10.1038/s41598-023-33782-z (PMC10170159; doi:10.1038/s41598-023-33782-z)
Supplement: Supplementary file 1 — Supplementary Information. [file 41598_2023_33782_MOESM1_ESM.pdf]

Supplementary Information for:

Usefulness of the BACES Score in Nontuberculous Mycobacterial Pulmonary Disease for Various Clinical Outcomes

Hyung-Jun Kim, Myung Jin Song, Byoung Soo Kwon, Yeon Wook Kim, Sung Yoon Lim, Yeon-Joo Lee, Jong Sun Park, Young-Jae Cho, Choon-Taek Lee, and Jae Ho Lee

Supplementary Table S1. Definition of Deaths from Respiratory Cause or Infection

Deaths from respiratory cause or infection	Deaths not associated with respiratory cause or infection
Asthma	Acute myocardial infarction
Adult respiratory distress syndrome	Alzheimer's disease
Bacterial pneumonia, NEC	Angina pectoris
Bronchiectasis	Arterial embolism and thrombosis
Certain infectious and parasitic diseases	Amyloidosis
Hemorrhage from respiratory passages	Cardiac arrest
Other chronic obstructive pulmonary disease	Cerebral infarction
Other diseases of pulmonary vessels	Cholelithiasis
Other interstitial pulmonary diseases	Chronic ischemic heart disease
Pneumonia, organism unspecified	Chronic kidney disease
Pneumonitis due to solids and liquids	Complications and ill-defined descriptions of heart disease
Pyothorax	Diverticular disease of intestine
Respiratory failure, NEC	Essential(primary) hypertension
	Heart failure
	Injury, poisoning and certain other consequences of external causes(S00-T98)
	Intracerebral hemorrhage
	Leukemia of unspecified cell type
	Malignant neoplasm of bladder
	Malignant neoplasm of bronchus and lung
	Malignant neoplasm of colon
	Malignant neoplasm of hypopharynx
	Malignant neoplasm of liver and intrahepatic bile ducts
	Malignant neoplasm of esophagus
	Malignant neoplasm of other and ill-defined digestive organs
	Malignant neoplasm of other and

	unspecified parts of biliary tract<br><br>Malignant neoplasm of pancreas<br><br>Malignant neoplasm of prostate<br><br>Malignant neoplasm of stomach<br><br>Multiple myeloma and malignant plasma cell neoplasms<br><br>Myelodysplastic syndromes<br><br>Myeloid leukemia<br><br>Non-follicular lymphoma<br><br>Other and unspecified types of non-Hodgkin lymphoma<br><br>Other general symptoms and signs<br><br>Other ill-defined and unspecified causes of mortality<br><br>Other neoplasms of uncertain or unknown behavior of lymphoid, hematopoietic and related tissue<br><br>Other sudden death, cause unknown<br><br>Osteoporosis with pathological fracture<br><br>Parkinson's disease<br><br>Sequelae of cerebrovascular disease<br><br>Senility<br><br>Subarachnoid hemorrhage<br><br>Type 2 diabetes mellitus<br><br>Mature T/NK-cell lymphomas

Supplementary Table S2. Patient symptoms upon diagnosis of NTM-PD

Variables	Overall<br>N = 681	BACES 0-1<br>n = 286	BACES 2-3<br>n = 335	BACES 4-5<br>n = 60	*p*
Cough	245 (36.0)	94 (32.9)	130 (38.8)	21 (35.0)	0.303
Sputum	198 (29.1)	83 (29.0)	100 (29.9)	15 (25.0)	0.748
Hemoptysis	124 (18.2)	64 (22.4)	52 (15.5)	8 (13.3)	0.052
Dyspnea	94 (13.8)	24 (8.4)	54 (16.1)	16 (26.7)	<0.001
Chest pain	29 (4.3)	15 (5.2)	14 (4.2)	0 (0.0)	0.187
Fever	31 (4.6)	7 (2.4)	21 (6.3)	3 (5.0)	0.074
Weight loss	17 (2.5)	3 (1.0)	10 (3.0)	4 (6.7)	0.029
Myalgia	4 (0.6)	0 (0.0)	4 (1.2)	0 (0.0)	0.125
Anorexia	3 (0.4)	0 (0.0)	1 (0.3)	2 (3.3)	0.002

Numbers are presented as count (percentage) or median [interquartile range]. Abbreviations: BMI, body mass index; IPF, idiopathic pulmonary fibrosis; COPD, chronic obstructive pulmonary disease.

Supplementary Table S3. Causative species of NTM among patients

Variables	Overall<br>N = 681	BACES 0-1<br>n = 286	BACES 2-3<br>n = 335	BACES 4-5<br>n = 60	*p*
					0.008
Mycobacterium avium complex	469 (68.9)	200 (69.9)	225 (67.2)	44 (73.3)	
M. avium	258 (37.9)	126 (44.1)	119 (35.5)	13 (21.7)	
M. intracellulare	181 (26.6)	56 (19.6)	94 (28.1)	31 (51.7)	
M. avium complex, unspecified	30 (4.4)	18 (6.3)	12 (3.6)	0 (0.0)	
Mycobacterium abscessus complex	98 (14.4)	44 (15.4)	44 (13.1)	10 (16.7)	
M. abscessus	76 (11.2)	32 (11.2)	36 (10.7)	8 (13.3)	
M. massiliense	20 (2.9)	11 (3.8)	7 (2.1)	2 (3.3)	
M. abscessus complex, unspecified	2 (0.3)	1 (0.3)	1 (0.3)	0 (0.0)	
M. kansasii	17 (2.5)	4 (1.4)	12 (3.6)	1 (1.7)	
M. fortuitum	5 (0.7)	0 (0.0)	4 (1.2)	1 (1.7)	
M. lentiflavum	5 (0.7)	3 (1.0)	2 (0.6)	0 (0.0)	
M. chelonae	1 (0.1)	0 (0.0)	1 (0.3)	0 (0.0)	
M. conceptionense	1 (0.1)	0 (0.0)	1 (0.3)	0 (0.0)	
M. gordonae	1 (0.1)	1 (0.3)	0 (0.0)	0 (0.0)	
M. septicum	1 (0.1)	0 (0.0)	1 (0.3)	0 (0.0)	
M. szulgai	1 (0.1)	0 (0.0)	1 (0.3)	0 (0.0)	
Mixed	55 (8.1)	24 (8.4)	27 (8.1)	4 (6.7)	
Unknown	27 (4.0)	10 (3.5)	17 (5.1)	0 (0.0)	

Numbers are presented as count (percentage). Chi-square test was used to obtain the *P*-value.

Supplementary Table S4. Details of the cause of death in NTM-PD patients

Variables	Overall<br>N = 209	BACES 0-1<br>n = 22	BACES 2-3<br>n = 140	BACES 4-5<br>n = 47
**Deaths by Respiratory cause or infection**				
Certain infectious and parasitic diseases (A00-B99)	29 (13.9)	6 (27.3)	15 (10.7)	8 (17.0)
Pneumonia, organism unspecified	16 (7.7)	0 (0.0)	11 (7.9)	5 (10.6)
Other chronic obstructive pulmonary disease	15 (7.2)	0 (0.0)	11 (7.9)	4 (8.5)
Other interstitial pulmonary diseases	13 (6.2)	1 (4.5)	11 (7.9)	1 (2.1)
Bronchiectasis	8 (3.8)	2 (9.1)	4 (2.9)	2 (4.3)
Asthma	4 (1.9)	0 (0.0)	2 (1.4)	2 (4.3)
Bacterial pneumonia, NEC	2 (1.0)	0 (0.0)	1 (0.7)	1 (2.1)
Pneumonitis due to solids and liquids	2 (1.0)	0 (0.0)	2 (1.4)	0 (0.0)
Adult respiratory distress syndrome	1 (0.5)	0 (0.0)	1 (0.7)	0 (0.0)
Haemorrhage from respiratory passages	1 (0.5)	0 (0.0)	1 (0.7)	0 (0.0)
Other diseases of pulmonary vessels	1 (0.5)	0 (0.0)	0 (0.0)	1 (2.1)
Pyothorax	1 (0.5)	0 (0.0)	1 (0.7)	0 (0.0)
Respiratory failure, NEC	1 (0.5)	0 (0.0)	1 (0.7)	0 (0.0)
**Deaths not caused by respiratory cause or infection**				
Malignant neoplasm of bronchus and lung	27 (12.9)	3 (13.6)	18 (12.9)	6 (12.8)
Malignant neoplasm of liver and intrahepatic bile ducts	8 (3.8)	0 (0.0)	7 (5.0)	1 (2.1)
Injury, poisoning and certain other consequences of external causes (S00-T98)	7 (3.3)	0 (0.0)	6 (4.3)	1 (2.1)
Acute myocardial infarction	5 (2.4)	0 (0.0)	4 (2.9)	1 (2.1)

Chronic kidney disease	4 (1.9)	1 (4.5)	3 (2.1)	0 (0.0)
Malignant neoplasm of pancreas	4 (1.9)	1 (4.5)	2 (1.4)	1 (2.1)
Senility	4 (1.9)	0 (0.0)	3 (2.1)	1 (2.1)
Angina pectoris	3 (1.4)	1 (4.5)	1 (0.7)	1 (2.1)
Cardiac arrest	3 (1.4)	0 (0.0)	2 (1.4)	1 (2.1)
Heart failure	3 (1.4)	1 (4.5)	2 (1.4)	0 (0.0)
Malignant neoplasm of colon	3 (1.4)	0 (0.0)	2 (1.4)	1 (2.1)
Malignant neoplasm of other and unspecified parts of biliary tract	3 (1.4)	1 (4.5)	1 (0.7)	1 (2.1)
Cholelithiasis	2 (1.0)	0 (0.0)	2 (1.4)	0 (0.0)
Chronic ischaemic heart disease	2 (1.0)	0 (0.0)	2 (1.4)	0 (0.0)
Malignant neoplasm of oesophagus	2 (1.0)	0 (0.0)	0 (0.0)	2 (4.3)
Malignant neoplasm of prostate	2 (1.0)	0 (0.0)	2 (1.4)	0 (0.0)
Multiple myeloma and malignant plasma cell neoplasms	2 (1.0)	0 (0.0)	2 (1.4)	0 (0.0)
Myelodysplastic syndromes	2 (1.0)	0 (0.0)	1 (0.7)	1 (2.1)
Myeloid leukaemia	2 (1.0)	0 (0.0)	2 (1.4)	0 (0.0)
Other general symptoms and signs	2 (1.0)	1 (4.5)	0 (0.0)	1 (2.1)
Parkinson’s disease	2 (1.0)	0 (0.0)	2 (1.4)	0 (0.0)
Alzheimer’s disease	1 (0.5)	1 (4.5)	0 (0.0)	0 (0.0)
Amyloidosis	1 (0.5)	0 (0.0)	1 (0.7)	0 (0.0)
Arterial embolism and thrombosis	1 (0.5)	0 (0.0)	1 (0.7)	0 (0.0)
Cerebral infarction	1 (0.5)	0 (0.0)	0 (0.0)	1 (2.1)
Complications and ill-defined descriptions of heart disease	1 (0.5)	0 (0.0)	1 (0.7)	0 (0.0)
Diverticular disease of intestine	1 (0.5)	0 (0.0)	1 (0.7)	0 (0.0)
Essential(primary) hypertension	1 (0.5)	0 (0.0)	1 (0.7)	0 (0.0)
Intracerebral haemorrhage	1 (0.5)	0 (0.0)	1 (0.7)	0 (0.0)
Leukaemia of unspecified cell type	1 (0.5)	0 (0.0)	1 (0.7)	0 (0.0)

Malignant neoplasm of bladder	1 (0.5)	0 (0.0)	1 (0.7)	0 (0.0)
Malignant neoplasm of hypopharynx	1 (0.5)	0 (0.0)	1 (0.7)	0 (0.0)
Malignant neoplasm of other and ill-defined digestive organs	1 (0.5)	0 (0.0)	0 (0.0)	1 (2.1)
Malignant neoplasm of stomach	1 (0.5)	0 (0.0)	1 (0.7)	0 (0.0)
Mature T/NK-cell lymphomas	1 (0.5)	1 (4.5)	0 (0.0)	0 (0.0)
Non-follicular lymphoma	1 (0.5)	0 (0.0)	1 (0.7)	0 (0.0)
Osteoporosis with pathological fracture	1 (0.5)	0 (0.0)	1 (0.7)	0 (0.0)
Other and unspecified types of non-Hodgkin lymphoma	1 (0.5)	1 (4.5)	0 (0.0)	0 (0.0)
Other ill-defined and unspecified causes of mortality	1 (0.5)	0 (0.0)	1 (0.7)	0 (0.0)
Other neoplasms of uncertain or unknown behaviour of lymphoid, haematopoietic and related tissue	1 (0.5)	0 (0.0)	1 (0.7)	0 (0.0)
Other sudden death, cause unknown	1 (0.5)	0 (0.0)	0 (0.0)	1 (2.1)
Sequelae of cerebrovascular disease	1 (0.5)	0 (0.0)	1 (0.7)	0 (0.0)
Subarachnoid haemorrhage	1 (0.5)	1 (4.5)	0 (0.0)	0 (0.0)
Type 2 diabetes mellitus	1 (0.5)	0 (0.0)	0 (0.0)	1 (2.1)

Numbers are presented as count (percentage).

Abbreviation: NEC, not elsewhere classified.

Supplementary Figure S1. Flowchart of Patient Selection Process

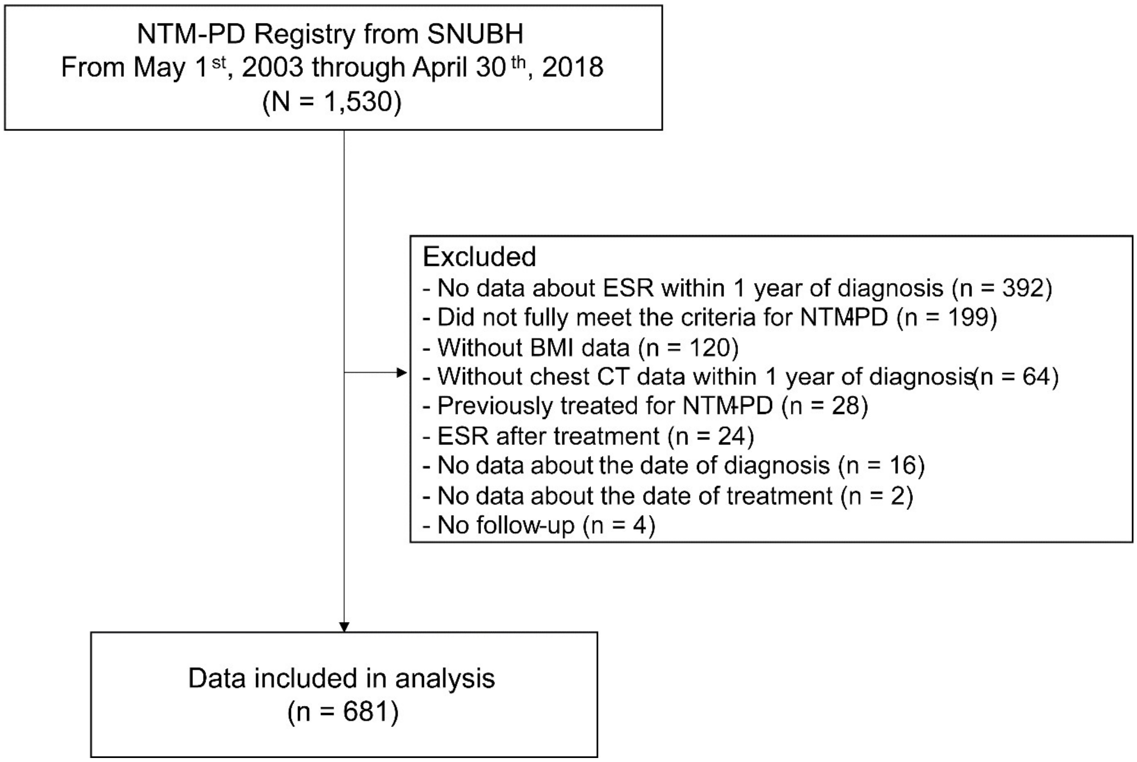

Abbreviations: NTM-PD, nontuberculous mycobacterial pulmonary disease; SNUBH, Seoul National University Bundang Hospital; ESR, erythrocyte sedimentation rate; BMI, body mass index; CT, computed tomography.

Supplementary Figure S2. Distribution of the BACES score

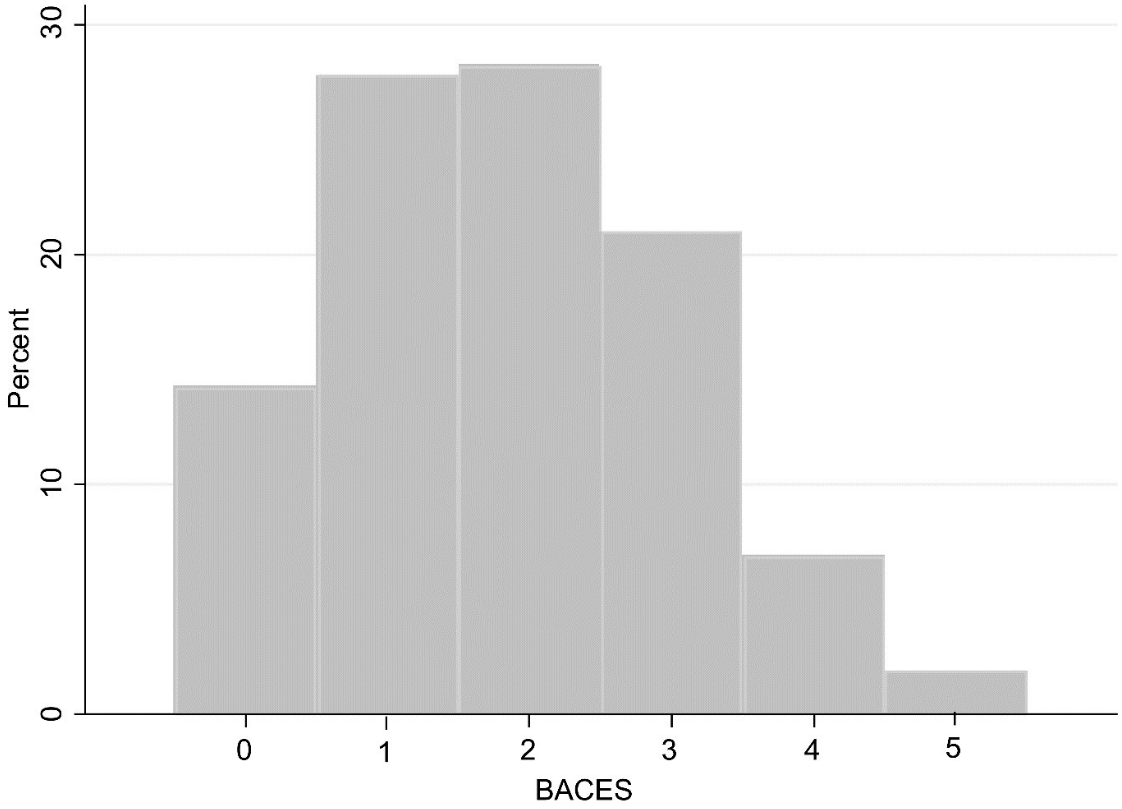

The distribution of the BACES score was as follows: BACES 0 (97 patients, 14.2%), BACES 1 (189 patients, 27.7%), BACES 2 (192 patients, 28.2%), BACES 3 (143 patients, 21.0%), BACES 4 (47 patients, 6.9%), and BACES 5 (13 patients, 1.9%).

Supplementary Figure S3. Calibration plot of all-cause survival probabilities according to three risk groups

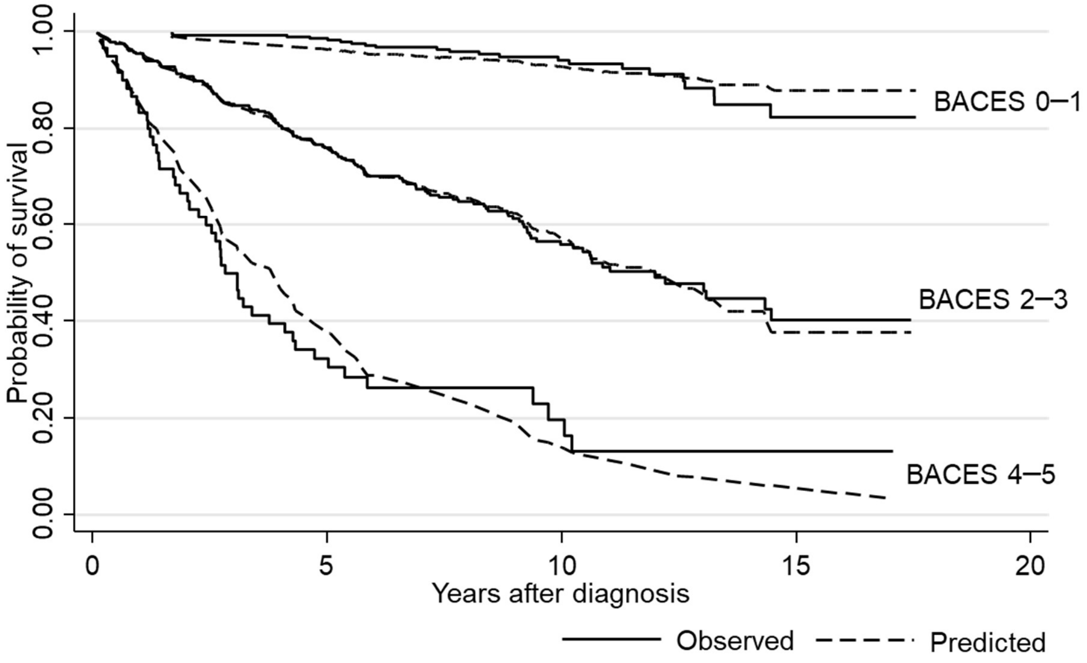

High (BACES 4-5), moderate (BACES 2-3), and low (BACES 0-1) risk groups are each represented. Kaplan-Meier estimations of the chance of survival are shown by solid lines. Estimated survival probabilities are shown by dashed lines using the Cox proportional hazards model.
